# Procyanidin A2, a polyphenolic compound, exerts anti-inflammatory and anti-oxidative activity in lipopolysaccharide-stimulated RAW264.7 cells

**DOI:** 10.1371/journal.pone.0237017

**Published:** 2020-08-05

**Authors:** Qin-Qin Wang, Hongwei Gao, Renyikun Yuan, Shan Han, Xin-Xing Li, Meiwen Tang, Baiqing Dong, Jun-Xiu Li, Li-Chun Zhao, Jianfang Feng, Shilin Yang

**Affiliations:** 1 College of Pharmacy, Guangxi University of Chinese Medicine, Nanning, China; 2 Guangxi Engineering Technology Research Center of Advantage Chinese Patent Drug and Ethnic Drug Development, Nanning, China; 3 College of Public Health and Management, Guangxi University of Chinese Medicine, Nanning, China; Chang Gung University, TAIWAN

## Abstract

Procyandin A2 (PCA2) is a polyphenolic compound which is isolated from grape seeds. It has been reported that PCA2 exhibits antioxidative and anti-inflammatory effects, but its molecular mechanism is still poorly understood. This study tests the hypothesis that PCA2 suppresses lipopolysaccharide (LPS)-induced inflammation and oxidative stress through targeting the nuclear factor-κB (NF-κB), mitogen-activated protein kinase (MAPK), and NF-E2-related factor 2 (Nrf2) pathways in RAW264.7 cells. PCA2 (20, 40, 80 μM) exhibited no significant cytotoxicity in RAW264.7 cells and showed an inhibitory effect on an LPS-induced nitrite level. Pro-inflammatory cytokines like tumor necrosis factor-α (TNF-α), interleukin-6 (IL-6), prostaglandin E2 (PGE2), nitric oxide (NO), and reactive oxygen species (ROS) were suppressed by PCA2 with a concentration range of 0–80 μM. The mRNA levels of TNF-α and IL-6 were inhibited by PCA2 (80 μM). The hallmark-protein expression of the NF-κB (p-IKKα/β, p-IκBα, and p-p65) and MAPK (p-p38, p-JNK, and p-ERK) pathways were decreased by PCA2 in LPS-stimulated RAW264.7 cells. In addition, immunofluorescence results indicated that PCA2 (80 μM) promoted the translocation of NF-κB/p65 from the cytoplasm into the nucleus. PCA2 upregulated the expressions of Nrf2 and HO-1 and downregulated the expression of Keap-1. Simultaneously, PCA2 (80 μM) reversed LPS-induced Nrf2 translocation from the nucleus into the cytoplasm. Collectively, PCA2 protect cells against the damage from inflammation and oxidative injury, which suggest a potential therapeutic strategy for inflammatory and oxidative stress through targeting NF-κB, MAPK, and Nrf2 pathways in RAW264.7 cells.

## Introduction

Inflammation is a response when the body is stimulated by biological factors (bacteria, parasites), physical factors (high temperature, radioactive substances), chemical factors (chemical substances, endogenous toxins), foreign bodies (metals, wood chips) and other irritants [1, 2]. Previous research has shown that inflammation is a defensive response of the body [3]. The purpose of initial inflammation is to eliminate damage factors, promote the healing of damaged tissues as well as regeneration of necrotic tissue so that the pathogenic factors were limited to the site of inflammation and does not spread throughout the whole body [4, 5]. However, accucmulating evidence indicates that inflammation results in numerous diseases such as arthritis, rheumatoid arthritis, bowel inflammatory disease (BID), etc. [6, 7].

Inflammatory processes include vascular reactions and white blood cell reactions, which are achieved through the action of a series of chemical factors [8]. A monocyte/macrophage-like cell line plays a valuable role in the inflammatory process [9]. Thus, RAW264.7 macrophage is the most generally employed for drug screening for anti-inflammatory activity *in vitro* [9]. LPS, toll-like receptor 4 (TLR4) agonist, induces inflammation response on the macrophage, which induces pro-inflammatory cytokines release [10, 11]. During pathological inflammation, immune cells are activated first [12]. Afterwards, the cells are collected to the injured area, caused cytokines like TNF-α, IL-6 release and the generation of reactive oxygen species (ROS), which damages tissue-repair [13]. In keep with this, iNOS and COX-2 will overexpress, leading to NO and PGE2 release, respectively, to activate multiple immune-pathological pathways [14]. Nuclear factor (NF-κB) can specifically bind to a variety of promoters to promote its transcriptional expression. After being stimulated by multiple factors, it can regulate the production of pro-inflammatory factors [15]. NF-κB is consists of IκB, p65, and p50. When NF-κB is quiescent, IκB and NF-κB polymerization remains in the cytoplasm as a trimeric form [16]. However, after stimulation by external signals such as LPS, the IKK complex is activated, which in turn causes IKB phosphorylated, and NF-κB released from cytoplasm to the nucleus, thereby activating the corresponding gene’s expression [17]. Apart from the NF-κB pathway, the MAPKs pathway is one of the important ways in cell signal transmission to be pivotal in the inflammatory process. MAPKs involving ERK, JNK, and p38, activates transcription factors-1 to mediate some inflammatory factors expression [18]. Consequently, the NF-κB and MAPK are classic signaling pathways, which regulate inflammation signal transmission. In addition, oxidative stress is closely associated with the occurrence of various diseases [19]. The Nrf2 pathway improves the body’s oxidative stress state via regulating the expression of oxidized proteins, promoting cell survival and maintaining the cell’s redox homeostasis, thereby protecting the body from damage [20, 21]. Generally, Nrf2 and Keap1 are combined in the cytoplasm. If it has not been activated, Nrf2 will be ubiquitinated and degraded [22]. The binding of Keap1and Nrf2 becomes unstable when oxygen free radicals come into being. Meanwhile, Nrf2 is released and transferred to the nucleus, where it combines with ARE and activates the downstream genes to translate a series of related proteins (such as HO-1) for physiological functions [23]. Thus, through regulating Nrf2 nucleus translocation to increase HO-1 activity, the body can be protected from oxidative stress damage.

**Fig 1:**
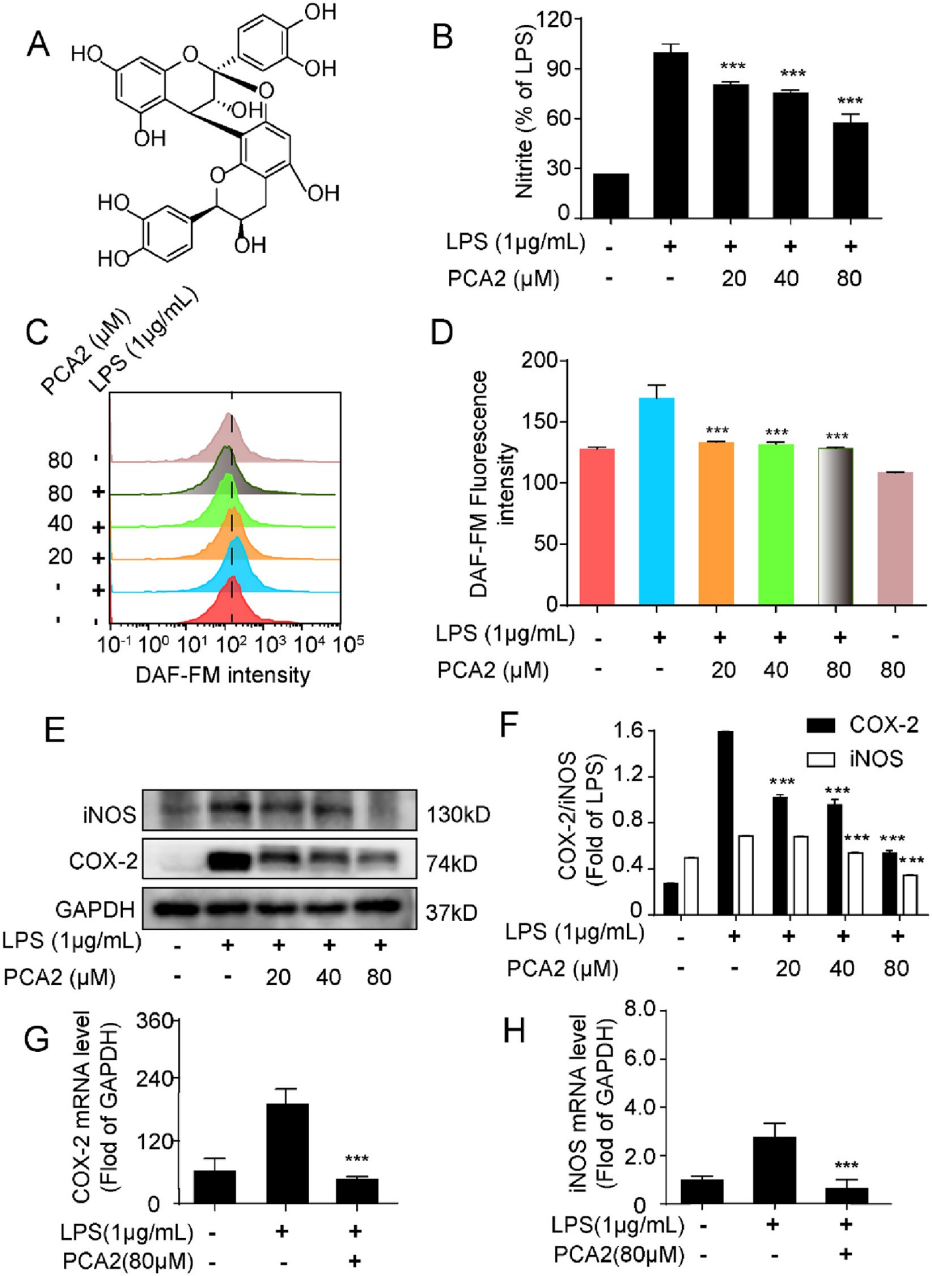
PCA2 suppressed LPS-induced macrophage activation. **(A)** The chemical structure of PCA2. **(B)** Cells were cultured with LPS for 18 h after treated with PCA2 (20, 40, 80 μM) for 1 h, except for the control group. Determination of nitrite levels by the Griess assay (n = 5). **(C)** Cells were cultured with LPS for 6 h after treated with PCA2 (20, 40, 80 μM) for 1 h, except the control group. After cells were incubated with DAF-FM (1 μM) for 1 h, collected to flow tube and monitored by the flow cytometry (n = 3). (**D**) Statistical analysis fluorescence intensity of DAF-AM (n = 3). (**E**) Cells were cultured with LPS for 18 h after treated with PCA2 (20, 40, 80 μM) for 1 h, except for the control group. iNOS and COX-2 protein expression were tested by western blotting (n = 3). (**F**) Statistical analysis of the expression of the protein (n = 3). (**G**, **H**) Cells were cultured with LPS for 2 h after treated with PCA2 (80 μM) for 1 h, except for the control group. The mRNA expression of COX-2 and iNOS tested by qRT-PCR (n = 3). ***p < 0.001 vs. LPS group.

Procyanidin, polyphenolic secondary metabolites, is a compound formed by the polymerization of flavan-3-ol as a structural unit through C-C bond, and its mechanism is currently unclear [24]. Procyanidin A2 (PCA2) is an A-type proanthocyanidin dimer formed by the polymerization of epicatechin (Fig 1A) and preponderantly locate in the nucleus, shell, seed, flower, and leaves of many plants [24]. Specifically, the content of PCA2 is occupies a large percentage in grape seeds [25]. In addition to PCA2, many other pure compounds such as procyanidin A1, A2, B1-B8, C1, and C2 were isolated and identified [26]. According to a comprehensive review of procyanidins, procyanidins exhibited multiple bio-activities like anti-oxidative, anti-inflammatory, antidiabetic, antimicrobial cardioprotective, neuroprotective, immunomodulatory, and lipid-lowering and anti-obesity activities [26]. Whereas, most of the studies mainly focused on the mixture of procyanidins. And B-type proanthocyanidins are bind together via C4β→C8, C4β→C6, C4α→C8 or C4α→C6 bonds, while A-type proanthocyanidins have another interflavan bond C2β→O7, and procyanidins A1, A2, B1, B2, B3 are dimers, procyanidin C1 is trimer [27]. Increasing evidence has demonstrated that procyanidin A1 has anti-inflammatory and anti-oxidative effects on LPS-stimulated RAW264.7 cells [9]. Procyanidin B1, B2, and C1 showed anti-inflammatory and oxidative activities in human monocytes [28]. In addition, procyanidin B2 inhibited NLRP3 inflammasome activation in human vascular endothelial cells [29]. A previous study indicated that PCA2 has anti-oxidative and anti-inflammatory effects. Nevertheless, the mechanism of PCA2’s anti-oxidative and anti-inflammatory activities is still needs further investigation. Accordingly, in this study, we evaluated the mechanism of PCA2’s anti-oxidative and anti-inflammatory activities in RAW264.7 cells.

## Materials and methods

### Materials

Procyanidin A2 was acquired from PUSH BIO-TECHNOLOGY (Chengdu, China). The purity of PCA2 (over 95%) was determined by HPLC. Griess reagent (modified-G4410), Indomethacin was purchased from MACKIN (#C10429693). Lipopolysaccharides from *Escherichia coli* O111:B4. DCFH_2_-DA were purchased from Sigma-Aldrich (St. Louis, MO, USA). PGE2 ELISA kit was bought from Caymanchem (Ann Arbor, MI, USA). IL-6 and TNF-α ELISA kits were purchased from Neonbioscience (Shenzhen, China). Antibodies: the NF-κB pathway sampler kit (#8242T), p-NF-κB/p65 (#3033), IKKα (#2682), IKKβ (#8943T), p-IKKα/β (#2078), IκBα (#4814), p-IκBα (#2859), iNOS (#13120), COX-2 (#4842); the MAPK pathway sampler kit: JNK1/2 (#9252T), p-JNK1/2 (#4668T), ERK1/2 (#4695T), p-ERK1/2 (#4370T), p38MAPK (#9212), p-p38MAPK (#4511); the Nrf2 pathway sampler kit: Keap-1 (#4678), Nrf2 (#12721), HO-1 (#70081), PARP (#9532) and GAPDH (#5174) were obtained from Cell Signaling Technologies (Beverly, MA, USA). Dulbecco’s modified eagle’s medium (DMEM), Fluo-3/AM, DAF-FM, and fetal bovine serum (FBS) were obtained from Life Technologies/Gibco Laboratories (Grand Island, NY, USA). MAK-143 intracellular ROS kit, dimethyl sulfoxide (DMSO) were acquired from Sigma-Aldrich (St. Louis, MO, USA). Dual-Glo luciferase assay system kit and qRT-PCR kit were bought from Thermo Fisher (Waltham, MA, USA). Nuclear and Cytoplasmic Protein Extraction kit was purchased from Beyotime (Shanghai, China).

### Cell culture

The RAW264.7 cell line was bought from the Cell Bank of the Chinese Academy of Sciences (Shanghai, China). They were cultured in DMEM medium containing 10% FBS, 1% Penicillin/Streptomycin and kept in a humidified air with 5% CO_2_ at 37 °C. When the cells grow at a fusion degree of about 70–80%, they are subcultured. Straight after, collect the cells with cells gently in a centrifuge tube to centrifuge (800 rpm, 5 min). Subsequently, discard the supernatant and add 1 mL medium, blow up the cells with a pipettor. Finally, put the cells in culture flask and place them in the incubator. Collect the cell’s pellets in the logarithmic growth phase, and adjust the cell density according to the requirements of the number of cells in the seed plate before further treatments.

### Cell viability assay

Cells were cultured in 96-well plates, containing 5×10^5^ cells in each well, for 14 h. Then, they were treated with PCA2 for 24 h. Subsequently, the MTT solution (5 mg/mL) was added to each well and cultured for 4 h. After then, discarded the culture media and add DMSO (100 μL/well). Until the purple crystals solved completely then measured absorbance at 570 nm using a microplate reader (BioTek, Winkowski, VT, USA).

### Determination of nitrite, IL-6, TNF-α, and PGE2

Cells were cultured in 24-well plates, containing 1×10^5^ cells in each well, for 14 h. Then cells were cultured with LPS (1 μg/mL) for 18 h before treated with PCA2 (20, 40, 80 μM) for 1 h, except the control group. The nitrite level was determined by Griess reagent. And the quantitative determination of IL-6, TNF-α, and PGE2 by ELISA kits according to the manufacturers’ instructions.

### Determination of reactive oxygen species (ROS) and NO

According to our previous protocol [12], cells were cultured in 24-well plates, containing 1×10^5^ cells in each well, for 14 h. To find out the maximum ROS generation time after LPS stimulated, LPS was used to induce cells for 0, 2, 4, 8, 10 h, later, cells were incubated with DCFH2-DA (1 μM, 0.5 h), collected to flow tube and monitored by the flow cytometry (Becton-Dickinson, Oxford, UK). Then, cells were co-cultured with LPS for 8 h or 6 h before treated with PCA2 (20, 40, 80 μM) for another 1 h, except the control group and only treated with PCA2 group. What’s more, DCFH2-DA and DAF-FM were employed to label cells, respectively to detect ROS level, NO level. After 0.5 h or 1 h, cells were incubated with DCFH2-DA (1 μM) or and DAF-FM (1 μM), collected to flow tube and monitored by the flow cytometry.

Cells were cultured in 96-well plates, containing 1×10^5^ cells in each well, for 14 h. Then, the level of intracellular ROS was measured by the MAK-143 intracellular ROS kit according to the manufacturer’s instructions. Following, we collected supernatant in a new 96-well plate and fluorescence intensity measured by a fluorescence microplate reader.

Cells were cultured in 96-well plates, containing 1×10^5^ cells in each well, for 14 h. Then the cell was exposed to PCA2 (80 μM) for 1 h and cultured with LPS (1 μg/mL) for 8 h, except the control group. For the measurement of ROS, DCFH2-DA (1 μM) was loaded for 0.5 h and images were acquired with a fluorescence microscope (Leica, Wetzlar, Germany).

### Determination of mitochondrial membrane potential (MMP) and calcium level

According to our previous protocol [12], cells were cultured in 96-well plates, containing 1×10^5^ cells in each well, for 14 h. Then the cell was exposed to PCA2 (80 μM) for 1 h and cultured with LPS for 8 h, except the control group. For the measurement of MMP, JC-1 (10μg/mL) was loaded for 0.5 h and images were acquired with a fluorescence microscope.

Cells were cultured in 24-well plates, containing 2×10^5^ cells in each well, for 14 h. Then cells were cultured with LPS for 8 h before treated with PCA2 (20, 40, 80 μM) for 1 h, except control group and only treated with PCA2 group. Fluo-3/AM was employed to stain Ca^2+^. Then cells were incubated with Fluo-3/AM (1 μM) for 1 h, collected to flow tube and monitored by the flow cytometry.

### Immunofluorescence assay

According to our previous protocol [30], cells were cultured on confocal dishes, containing 2×10^5^ cells, for 14 h. The LPS-induced group cells were exposed to LPS (1 μg/mL) for 2 h, while the experiment group cells were exposed to PCA2 (80 μM) for 1 h and cultured with LPS (1 μg/mL) for same times. Subsequently, cells incubated with NF-κB p65 or Nrf2 antibody and secondary antibody for 1 h independently. Later, we used Hoechst 33342 to stain nuclei for a quarter. Images were taken with the confocal laser scanning microscopy (Leica, Wetzlar, Germany).

### Western blot analysis

Cells were cultured in dishes with the diameter is 60 mm, containing 2×10^6^ cells, and collected after different treatments. Cell lysate, protein concentration determination, electrophoresis, and immunoblotting were performed according to our previous protocol. After lysing the cells with cell lysate and determining the deproteinized concentration, perform electrophoresis and electroporation. Following, incubated with primary antibodies and secondary antibody. Signals of chemiluminescence intensity were detected by ChemiDoc^™^ MP Imaging System (Bio-Rad, Hercules, CA, USA).

### Quantitative Real-Time PCR (qRT-PCR) assay

According to our previous protocol [12], cells were cultured in dishes with the diameter is 60 mm, containing 2×10^6^ cells, for 14 h. The LPS-induced group’s cells were induced by LPS for 2 h, while the experiment group’s cells were exposed to PCA2 (80 μM) or Indo (80 μM) for 2 h. Trizol assay kit and RNase-free water were used to extracted total RNA according to the manufacturers’ instructions. The PCR amplification of RNA (1 μL) performed by incorporating SYBR green within the qPCR master mix kit. The gene sequences are as following: IL-6-F: TCCAGTTGCCTTC TTGGGAC, IL-6-R: GTGTAATTAAGCCTCCGACTTG; TNF-α-F: TTCTGTCTACTGAACTTCGGGGTGATCGGTCC, TNF-α-R: GTATGAGATAGCAAATCGGCTGACGGTGTGGG; COX-2-F: TGAGTACCGCAAACGCTTCTC, COX-2-R: GTGTAATTAAGCCTCCGACTTG; iNOS-F: GGCAGCCTGTGAGACCTTTG, iNOS-R: GTGTAATTAAGCCTCCGACTTG; NF-κB/p65-F: GCACGGATGACAGAGGCGTGTATAAGG, NF-κB/p65-R: GGCGGATGATCTCCTTCTCTCTGTCTG, NRF2-F: AGCAGGACATGGAGCAAGTT, NRF2-R: TTCTTTTTCCAGCGAGGAGA; GAPDH-F: CATGACCACAGTCCATGCCATCAC, GAPDH-R: TGAGGTCCACCACCC TGTTGCTGT; HO-1-F: TCAGTCCCAAACCTCGCGGT, HO-1-R: GCTGTGCAGGTGTTAGCC.

### Statistical analysis

All results were independently repeated on different three days, and are calculated mean ± SD. One-way analysis of variance (ANOVA) and Dunn’s multiple-comparison tests were used to compare differences in more than two groups with GraphPad Prism 6.0 software. Differences between groups were considered significant at p < 0.05.

## Results

### PCA2 suppressed LPS-induced macrophage activation

**Fig 2:**
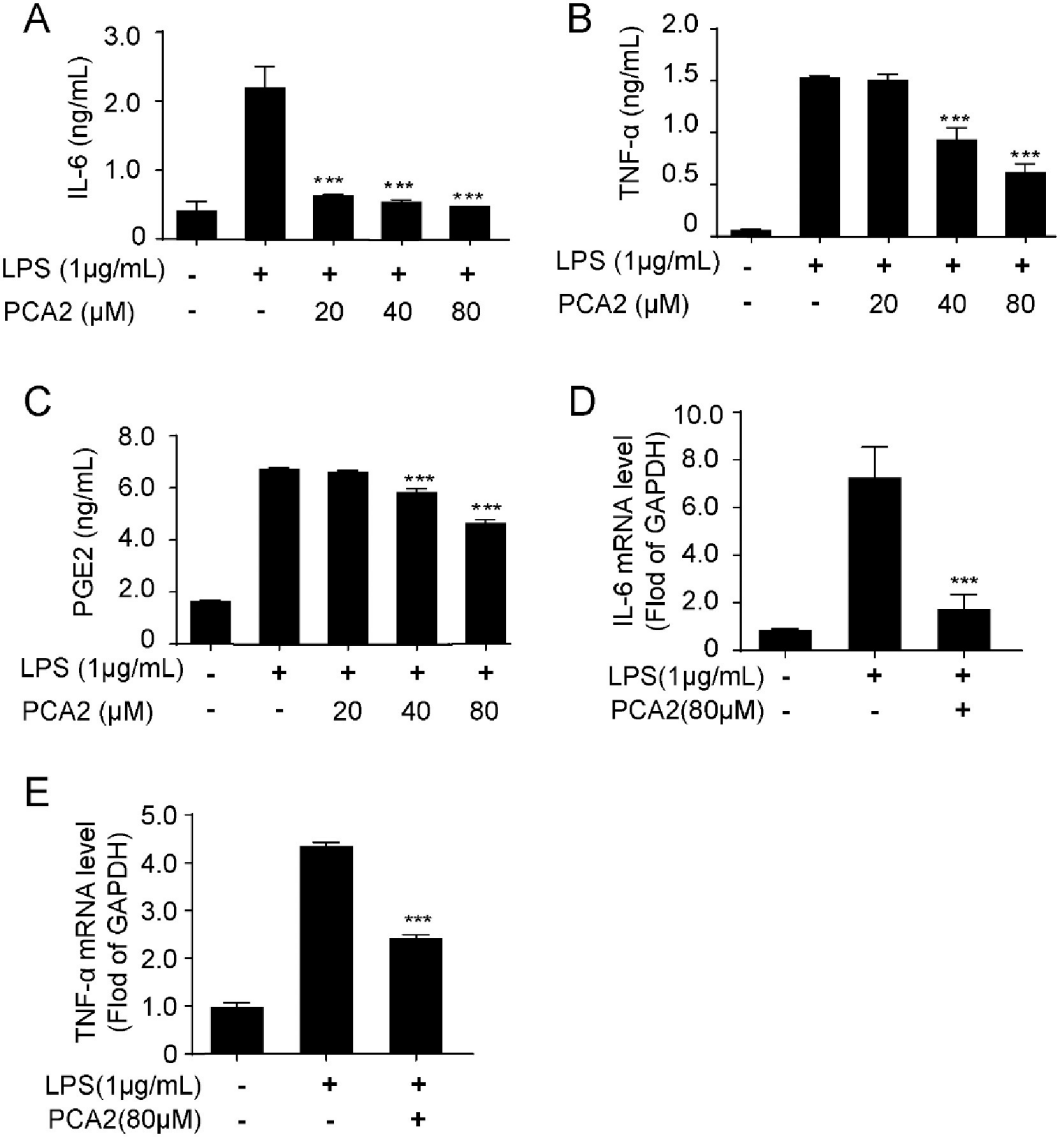
PCA2 inhibited pro-inflammatory cytokines release and gene expression. Cells were cultured with LPS for 18 h after treated with PCA2 (20, 40, 80 μM) for 1 h, except for the control group. (**A**) IL-6, (**B**) TNF-α, and (**C**) PGE2 were investigated by ELISA kits (n = 5). Cells were cultured with LPS for 2 h after treated with PCA2 (80 μM) for 1 h, except for the control group. The mRNA expression of (**D**) IL-6 and (**E**) TNF-αwere tested by qRT-PCR (n = 3). *p < 0.05, *p* < 0.01, ***p < 0.001 vs. LPS group.

Employing LPS-stimulated RAW264.7 cells, we studied whether PCA2 showed anti-inflammatory activity. Initially, the Griess reagent and the flow cytometry assays were used to detect nitrite and nitride oxide (NO) levels, respectively. Results indicated that PCA2 (20, 40, 80 μM) inhibited the increase of LPS-induced nitrite (Fig 1B) and NO levels (Fig 1C and 1D) in RAW264.7 cells. Furthermore, PCA2 (80 μM) not only restrained the iNOS and COX-2’s mRNA expression, but also the expression of the protein (Fig 1E–1H), the proteins expression and COX-2. Furthermore, to investigate the effect of PCA2 on proinflammatory cytokines release in LPS-induced RAW264.7 cells, we detected IL-6, TNF-α, and PGE2 secreted levels. Results showed that PCA2 (20, 40, 80 μM) decreased the release of IL-6, TNF-α, and PGE2 (Fig 2A–2C). Corresponding with the release of the cytokine, PCA2 (80 μM) suppressed mRNA expression of IL-6 and TNF-α (Fig 2D and 2E). PCA2 (20, 40, 80 μM) displayed no significant cytotoxicity (S1 Fig). Taken together, our findings indicated that PCA2 suppressed LPS-stimulated macrophage activation and displayed anti-inflammatory activity.

### PCA2 decreased LPS-induced reactive-oxygen-species generation

**Fig 3:**
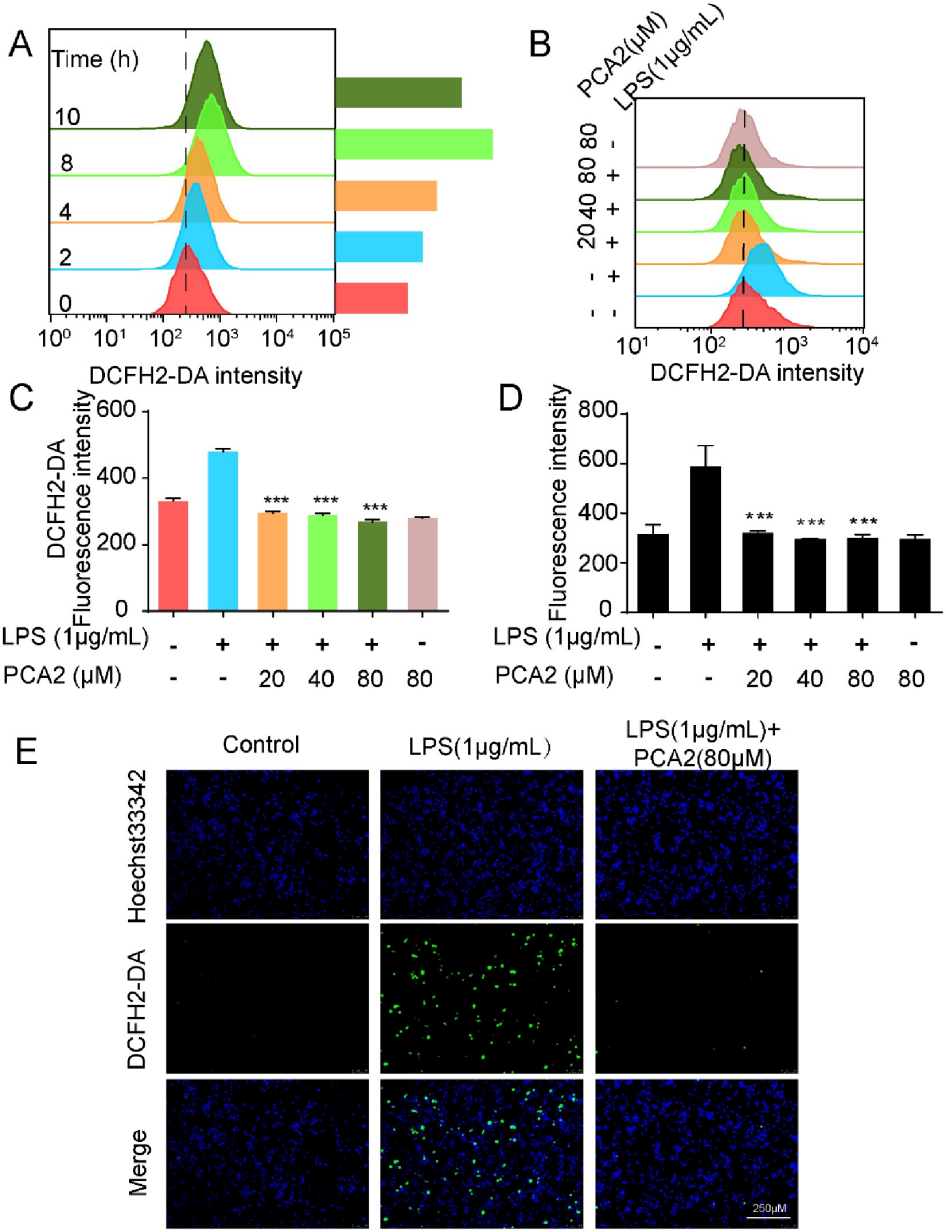
PCA2 decreased LPS-induced ROS generation. **(A)** LPS was used to induce cells for 0, 2, 4, 8, 10 h, and then cells were incubated with DCFH2-DA (1 μM) for 0.5 h, collected to flow tube and monitored by the flow cytometry (n = 3). **(B)** Cells were cultured with LPS for 8 h after treated with PCA2 (20, 40, 80 μM) for 1 h, except the control group and only treated with PCA2 group. After cells were incubated with DCFH2-DA (1 μM) for 0.5 h, collected to flow tube and monitored by the flow cytometry (n = 3). **(C)** Statistical analysis of the fluorescence intensity of DCFH2-DA (n = 3). (**D**) Cells were cultured with LPS for 8 h after treated with PCA2 (20, 40, 80 μM) for 1 h, except the control group and only treated with PCA2 group. Master Reaction Mix was placed into a cell plate and incubate for 1 h. Fluorescence intensity measured by a fluorescence microplate reader (n = 4). (**E**) The cell was exposed to PCA2 (80 μM) 1 h before cultured with LPS for 8 h, except for the control group. For the measurement of ROS, DCFH2-DA (10 μM) was loaded for 0.5 h and images were acquired with a fluorescence microscope (n = 3). ***p < 0.001 vs. LPS group.

Exhibited in Fig 3A, results showed that particularly induced ROS generation was particularly when LPS stimulated for 8 h stimulated. Then, we used the flow cytometry to test the effects of different concentrations of PCA2 (20, 40, 80 μM) on the ROS level after LPS stimulation for 8 h. Consequently, PCA2 (20, 40, 80 μM) could effectively inhibit LPS-stimulated ROS generation (Fig 3B and 3C). Using a MAK-143 intracellular ROS kit, we detected the ROS level of PCA2 treated RAW264.7 cells. Consistent with the flow cytometry results, PCA2 (20, 40, 80 μM) decreased LPS-induced ROS generation (Fig 3D). Besides, we observed ROS production by fluorescence microscopy. Results indicated that PCA2 (80 μM) decreased LPS-induced fluorescence intensity, which corresponds to previous results (Fig 3E). Collectively, our results corroborated that PCA2 cut down the level of ROS, suggesting that PCA2 displayed anti-oxidative activity.

### PCA2 reversed LPS-induced mitochondrial-membrane-potential (MMP) loss and calcium influx

**Fig 4:**
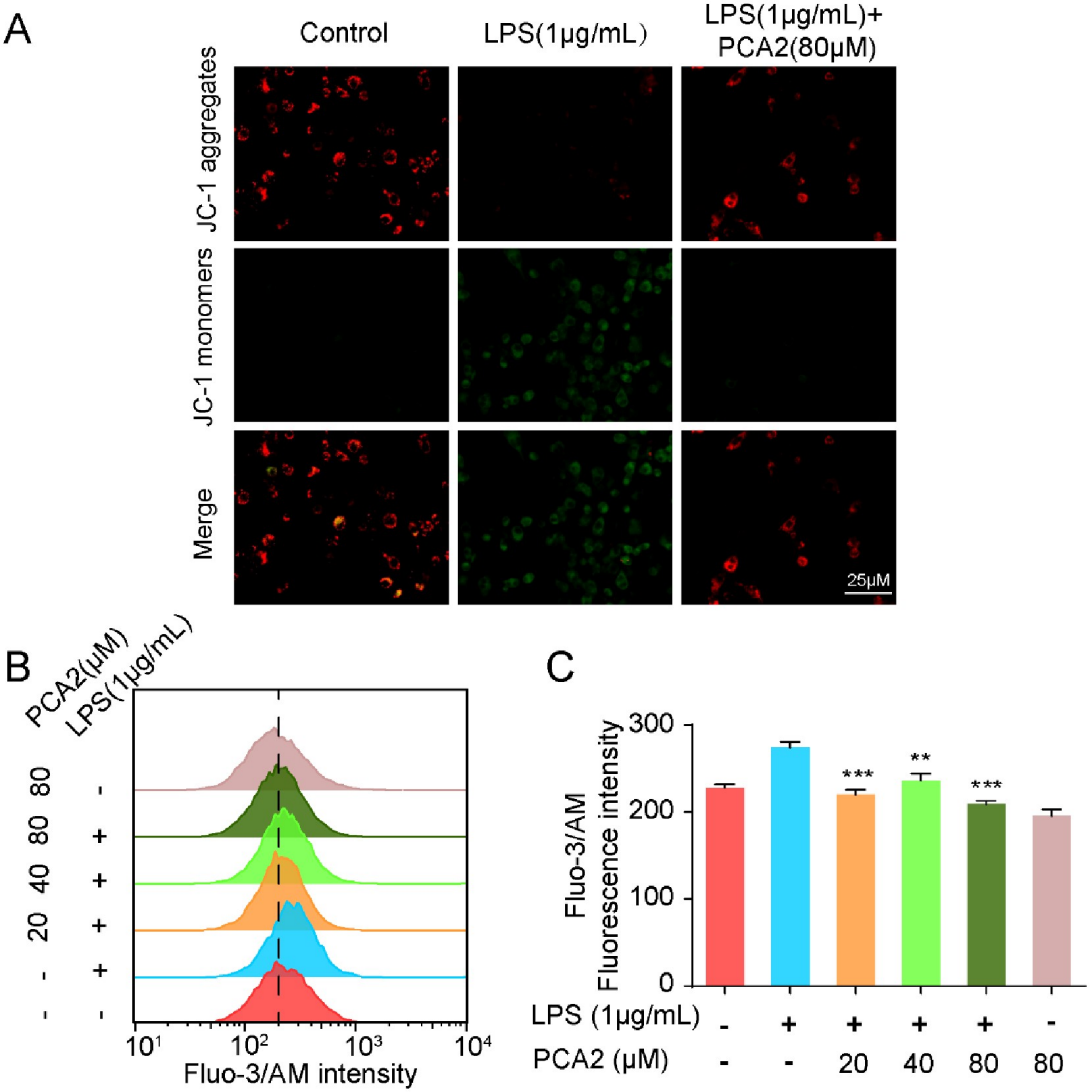
PCA2 reversed LPS-induced mitochondrial-membrane-potential (MMP) loss and calcium influx. **(A)** The cell was exposed to PCA2 (80 μM) for 1 h and cultured with LPS for 8 h, except for the control group. For the measurement of MMP, JC-1 (10 μg/mL) was loaded for 0.5 h and images were acquired with a fluorescence microscope (n = 3). **(B)** Cells were co-cultured with LPS for 6 h before treated with PCA2 (20, 40, 80 μM) for 1 h, except the control group and only treated with PCA2 group. After cells were incubated with Fluo-3/AM (1 μM) for 1 h, collected to flow tube and monitored by the flow cytometry (n = 3). (**C**) Statistical analysis of the fluorescence intensity of Fluo-3/AM (n = 3).**p < 0.01, *** *p* < 0.001 vs. LPS group.

The changes in mitochondrial membrane potential and calcium concentration are closely related to inflammatory diseases [31]. We observed changes in mitochondrial membrane potential by fluorescence biomicroscopy. Results indicated that under normal conditions, JC-1 in the cell mitochondria exists as a polymer, showing bright red fluorescence, and the mitochondrial membrane potential decreased after treatment with LPS, JC-1 cannot be present in the mitochondrial matrix as a polymer. The intensity of red fluorescence is significantly reduced, while the green fluorescence in the cytoplasm is significantly enhanced. After pretreatment with PCA2, this phenomenon was significantly reversed. It turns out that PCA2 (80 μM) rescued LPS-induced MMP loss (Fig 4A). Besides, we used flow cytometry to test the effects of PCA2 (20, 40, 80 μM) on calcium-level changes when LPS stimulated for 8 h. Results showed that PCA2 could effectively inhibit calcium influx (Fig 4B and 4C).

### PCA2 blocked NF-κB/p65 translocation

**Fig 5:**
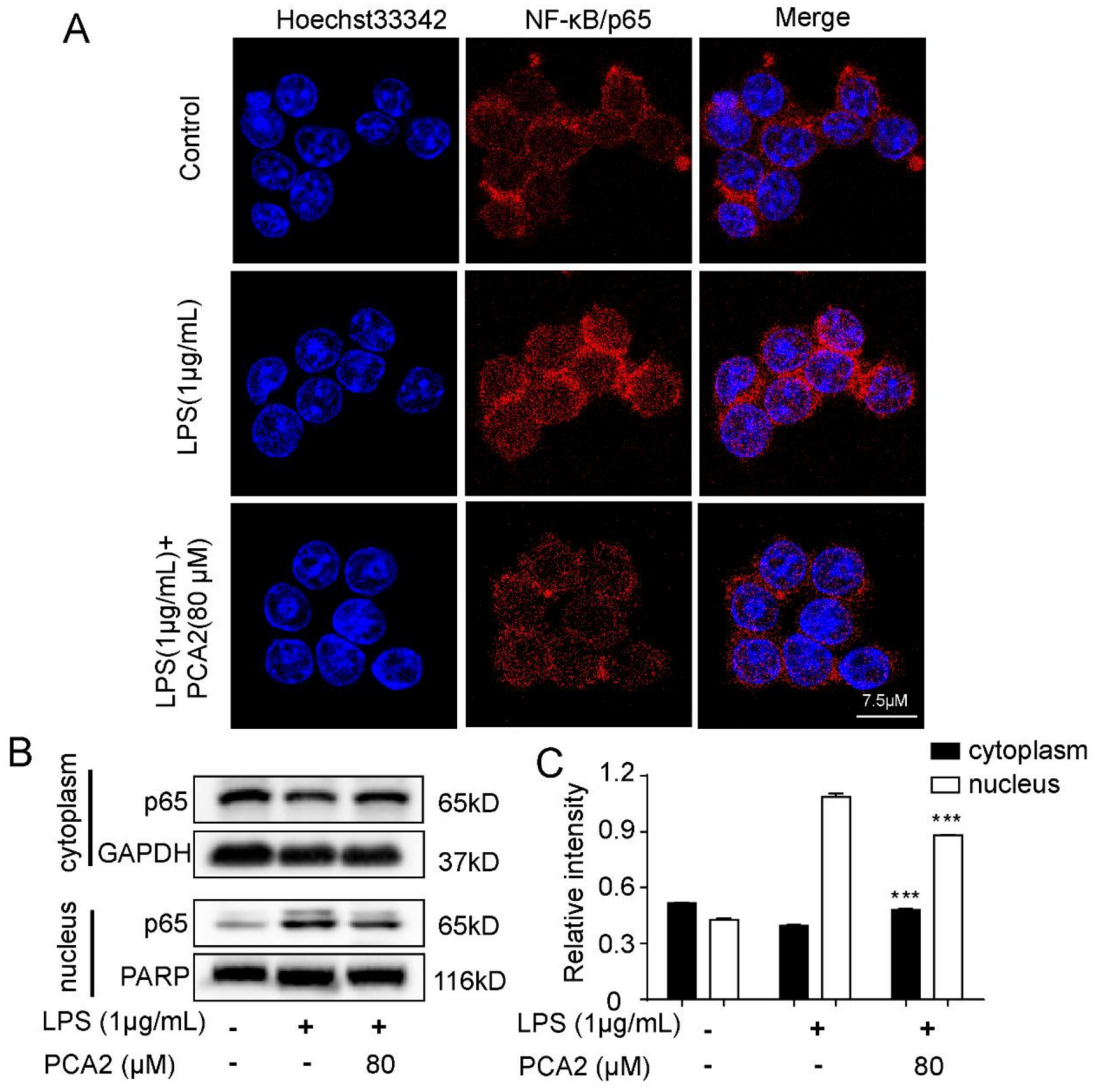
PCA2 blocked LPS-induced NF-κB/p65 translocation. **(A)** The LPS-induced group cells were exposed to LPS for 2 h, while the experiment group cells were exposed to PCA2 (80 μM) for 1 h and cultured with LPS for another 1 h. Subsequently, cells incubated with NF-κB p65 antibody and secondary antibody for 1 h independently. Later, we used Hoechst 33342 to stain nuclei for a quarter. Images were taken with the confocal laser scanning microscopy. **(B)** The cell was exposed to PCA2 (80 μM) for 1 h and cultured with LPS for 2 h, except for the control group. The protein expressions of p65 were detected by western blotting (n = 3). (**C**) Statistical analysis of the p65 protein expression in the cytoplasm and the nucleus (n = 3). *** *p* < 0.001 vs. LPS group.

Many inflammatory mediators will be released when NF-κB/p65 translocated [1]. Thus, the translocation of NF-κB/p65 is a marker of inflammation. Using laser confocal microscopy, we detected the translocation of NF-κB/p65. Experimental results showed that PCA2 (80 μM) inhibited LPS-induced p65 translocation (Fig 5A). Furthermore, the western blotting results demonstrated that PCA2 (80 μM) can reverse LPS-induced the exchange of NF-κB/p65 protein expression in cytoplasm and nucleus this was consistent with the immunofluorescence results (Fig 5B–5C). In conclusion, PCA2 suppressed LPS-induced NF-κB/p65 translocation.

### PCA2 ameliorated LPS-induced macrophage activation through the NF-κB/MAPK pathways

**Fig 6:**
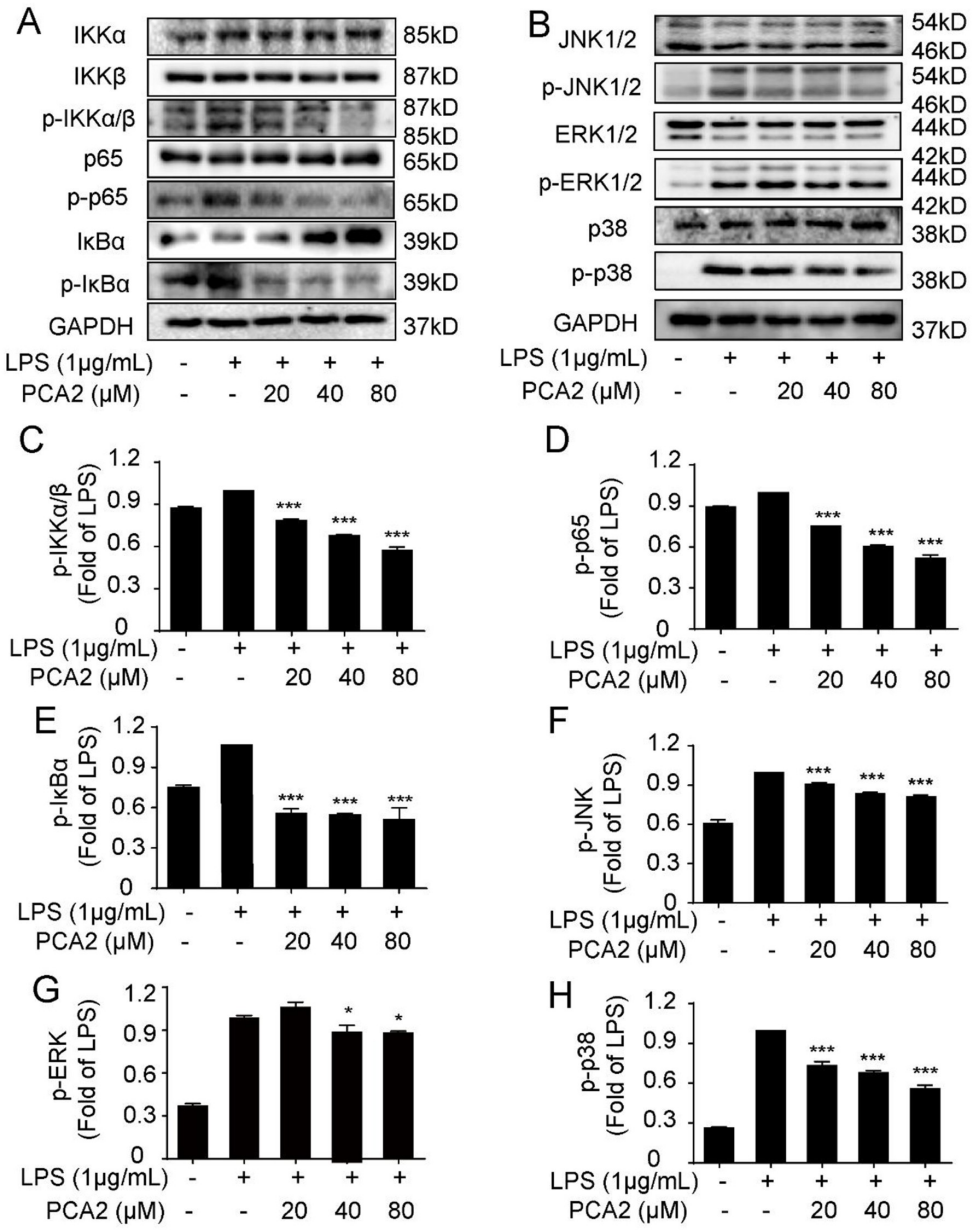
PCA2 ameliorated LPS-induced inflammatory activity through the NF-κB/MAPK pathways. The cell was exposed to PCA2 (20, 40, 80 μM) for 1 h and cultured with LPS for 8 h, except for the control group. Protein expression of **(A)** NF-κB and **(B)** MAPK pathway including **(C)** p-IKKα/β, **(D)** p-p65, **(E)** p-IκBα, **(F)** p- JNK1/2, **(I)** p-ERK1/2, and **(J)** p-p38 was measured by western blotting (n = 3). *p < 0.05, *** *p* < vs. LPS group 0.01.

To further research the anti-oxidative and anti-inflammatory mechanisms of PCA2 in LPS-induced RAW264.7 cells, we detected the expression of the protein on NF-κB and MAPK pathways by western blotting. Exhibited in Fig 6, PCA2 (20, 40, 80 μM) reduced the phosphorylation of IKK, p65, and IκBα (the NF-κB pathway) and JNK, ERK, and p38 (the MAPK pathway) without altering total proteins expression. In addition, we detected the NF-κB/p65 mRNA expression. Results suggested that PCA2 (80 μM) suppressed NF-κB/p65 mRNA expression. Taken collectively, PCA2 displays anti-inflammatory activity by regulating the NF-κB/MAPK pathways in RAW264.7 cells.

### PCA2 attenuated LPS-induced macrophage activation through the Nrf2 pathways

**Fig 7:**
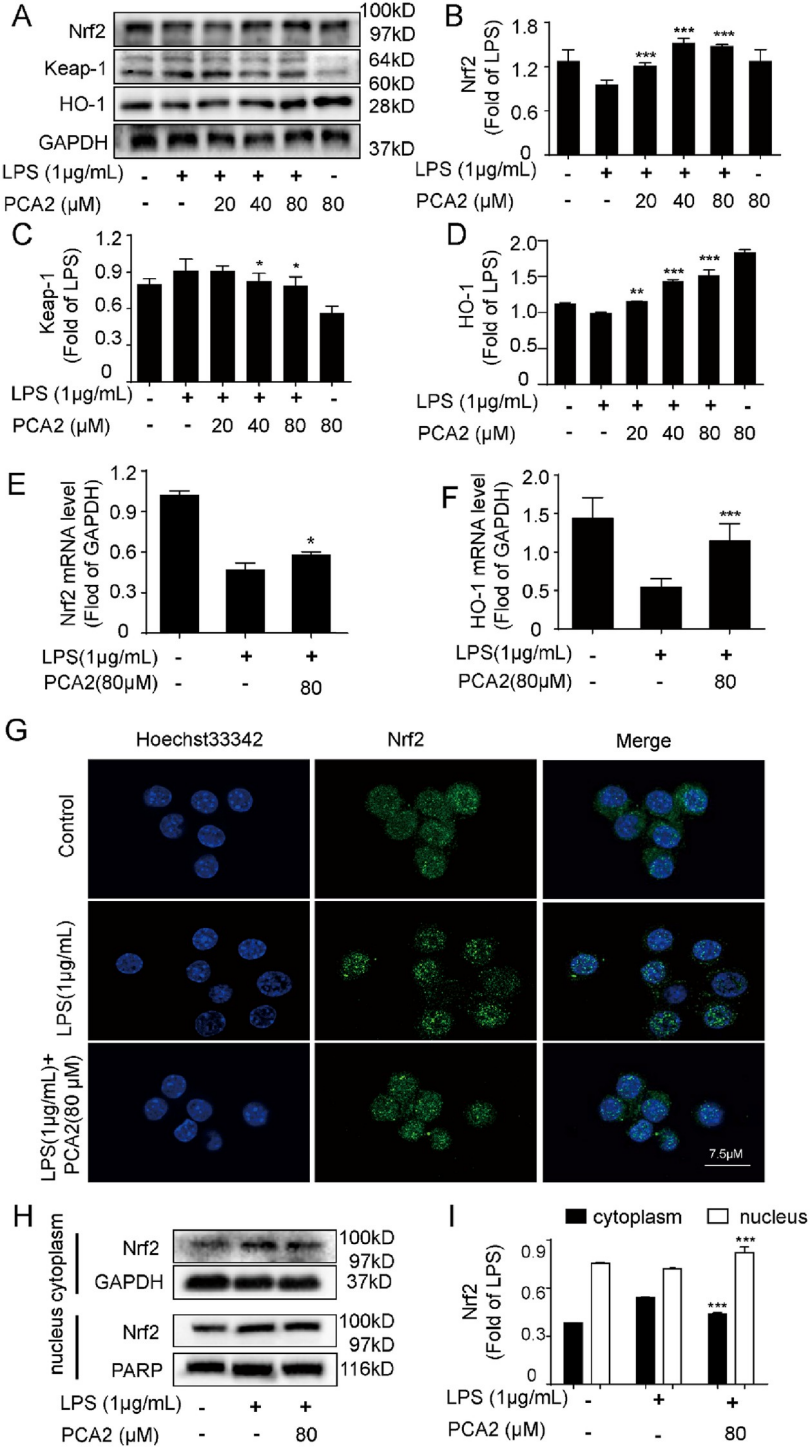
PCA2 attenuated LPS-induced inflammatory activity through Nrf2 pathways. The cell was exposed to PCA2 (20, 40, 80 μM) for 1 h and cultured with LPS for 8 h, except for the control group. The protein expression of the **(A)** Nrf2 pathway including **(B)** Nrf2, **(C)** Keap-1, **(D)** HO-1 was measured by western blotting. Cells were co-cultured with LPS for 2 h before treated with PCA2 (80 μM) for 1 h, except for the control group. **(E)** Nrf2, **(F)** HO-1 mRNA level was determined by qRT-PCR (n = 3). **(G)** The LPS-induced group cells were exposed to LPS for 2 h, while the experiment group cells were exposed to PCA2 (80 μM) for 1 h and cultured with LPS for another 1 h. Subsequently, cells incubated with Nrf2 antibody and secondary antibody for 1 h independently. Later, we used Hoechst 33342 to stain nuclei for a quarter. Images were taken with the confocal laser scanning microscopy. **(H)** The cell was exposed to PCA2 (80 μM) for 1 h and cultured with LPS for 2 h, except for the control group. The protein expressions of Nrf2 were detected by Western blotting (n = 3). **(I)** Statistical analysis of the Nrf2 protein expression in the cytoplasm and the nucleus (n = 3). *p < 0.05, *** *p* < 0.001 vs. LPS group.

The Nrf2 pathway is deemed to be extremely important in the antioxidant defense system [32]. Therefore, we detected related protein expression and mRNA expression. Results showed that LPS treatment pushed up Nrf2 and HO-1 protein expression, and decreased Keap-1 expression, which was reversed by PCA2 (20, 40, 80 μM) (Fig 7A–7D). In addition, the qRT-PCR results showed that PCA2 (80 μM) reversed Nrf2 and HO-1 mRNA expression (Fig 7E and 7F). Furthermore, we investigated the translocation of Nrf2 and the expression of nucleus and cytoplasm in RAW264.7 cells. Results revealed that PCA2 (80 μM) has the ability to promote Nrf2 translocation from the cytoplasm into the nucleus in LPS-induced RAW264.7 cells (Fig 7G–7I), meaning that PCA2 attenuated Nrf2 translocation from the nucleus into the cytoplasm. Taken together, our findings demonstrated that PCA2 exerted an anti-oxidative effect *via* the Nrf2 pathway.

## Discussion

The inflammatory response is related to the occurrence and development of many diseases, for instance of psoriasis, rheumatoid arthritis [33]. The main functions of activated macrophages are to engulf the cell debris and pathogens, and activate lymphocytes to make them respond to irritants [34]. On the other hand, LPS-stimulated macrophage is a classic model of inflammatory responses *in vitro* [33]. Previous studies revealed that PCA2 has the effects of protecting cells from oxidation, inflammation, such as human monocyte macrophages (THP-1) [26] and L0-2 cells [35], A549 cells [24], and BV2 cells [36], respectively. PCA2 exhibited anti-inflammatory activity *via* suppressing CCL-26 production in IL-4-stimulated A549 cells [24] and anti-oxidative activity *via* modulating the Nrf2 pathway in *tert*-butyl hydroperoxide-stimulated L0-2 cells [35]. PCA2 has a neuroprotective effect on Aβ-stimulated BV2 cells [36]. However, the detailed mechanism of PCA2’s anti-inflammatory and anti-oxidative activity is still uncertain. Here, employing the LPS-stimulated RAW264.7 cells model, we have revealed the mechanism of PCA2’s anti-inflammatory and anti-oxidative effects. We employed MTT assay to investigate PCA2’s cytotoxicity in RAW264.7 cells before determining the anti-inflammatory activity of PCA2. Results manifested that PCA2 at the concentration of 80 μM showed no detriment to cells (S1 Fig), which provides a guarantee of an anti-inflammatory effect of PCA2.

Under the stimulation of LPS, RAW26.7 cells activate inducible iNOS and COX enzyme systems, leading to cytokines like NO and PGE2 release [33]. Shan *et al* demonstrated that PCA1, an isomeride of PCA2, showed no effect on COX-2 and PGE2 in RAW264.7 cells [9]. However, in our study, PCA2 attenuated COX-2 expression and PGE2 release, which is different from PCA1 (Figs 1 and 2). Also, PCA2 not only reduced the protein expression of iNOS, but also the release of NO, TNF-α, and IL-6 (Figs 1 and 2). Taken together, our data suggested that PCA2 has an inhibitory effect on macrophage activation.

The generation of ROS not only mediates multifarious inflammatory signaling pathways but also promotes inflammatory response [37]. Excessive ROS negatively impacts mitochondria performance by decreasing MMP [38]. The protective effect of PCA2 on mitochondria was mainly achieved by inhibiting the generation of ROS and the loss of MMP loss indicated that it has a protective effect on mitochondria (Figs 3 and 4). The increase of intracellular Ca^2+^ in LPS-stimulated macrophages indicates that Ca^2+^ were involved in the activation of inflammation. We firstly found that the intracellular Ca^2+^ level was decreased after PCA2 pretreatment in RAW264.7 cells (Fig 4).

The DNA of NF-κB is a protein complex is present in nearly all animal cells, which covers cell growth and apoptosis [39]. During oxidative stress and inflammatory process, IκB kinase (IKK) activation leads to the phosphorylation of IκB and NF-κB’s translocation, suggesting that the NF-κB pathway is activated [40–42]. A previous study indicated that PCA2 exerted an anti-inflammatory effect in THP-1 cells *via* the NF-κB pathway [26]. In this study, employing RAW264.7 cells, we firstly found that PCA2 dramatically attenuated LPS-induced phosphorylation of NF-κB pathways proteins expression and nuclear translocation of p65 (Figs 5 and 6).

The MAPK pathway regulates various life processes of cells and participates in immune defense and inflammatory response [43–46]. As it turns out that PCA2 significantly decreased LPS-stimulated phosphorylation of MAPK pathway protein expression (Fig 6).

Nrf2, a member of the leucine regulatory protein family, is a critical transcription factor in the body’s regulation of oxidative stress [47, 48]. In general, Nrf2 binds to its repressor Keap-1, resulting in ubiquitination and subsequent degradation by the proteasome. However, upon exposure to oxidative stress, Nrf2 is translocated from the nucleus, and then interact with ARE to start the expression of the downstream anti-oxidant protein, for instance, HO-1 [49, 50]. On the one hand, HO-1 inhibits the production of pro-inflammatory cytokines, on the other hand, it also promotes the secretion of anti-inflammatory cytokines, thus achieving the purpose of anti-inflammatory [51]. Using a human fetal hepatocyte line (L-02), Hai-Yan Xu *et al* demonstrated that PCA2 against oxidative stress injury by initiated the Nrf2 pathway [35]. Employing RAW264.7 cells, we firstly showed that PCA2 pretreatment dramatically regulated the expression of the protein of the Nrf2 pathway *in vitro* (Fig 7).

In a general way, the Nrf2 signaling pathway inhibits the production cytokines and the inflammatory mediators, which affects the relevant pathways such as the NF-κB and MAPK pathway. Although we demonstrated that PCA2 exerted anti-oxidative and anti-inflammatory effects via the NF-κB, MAPK, and Nrf2 pathways, the molecular targets of PCA2 has not yet been illustrated.

**Fig 8:**
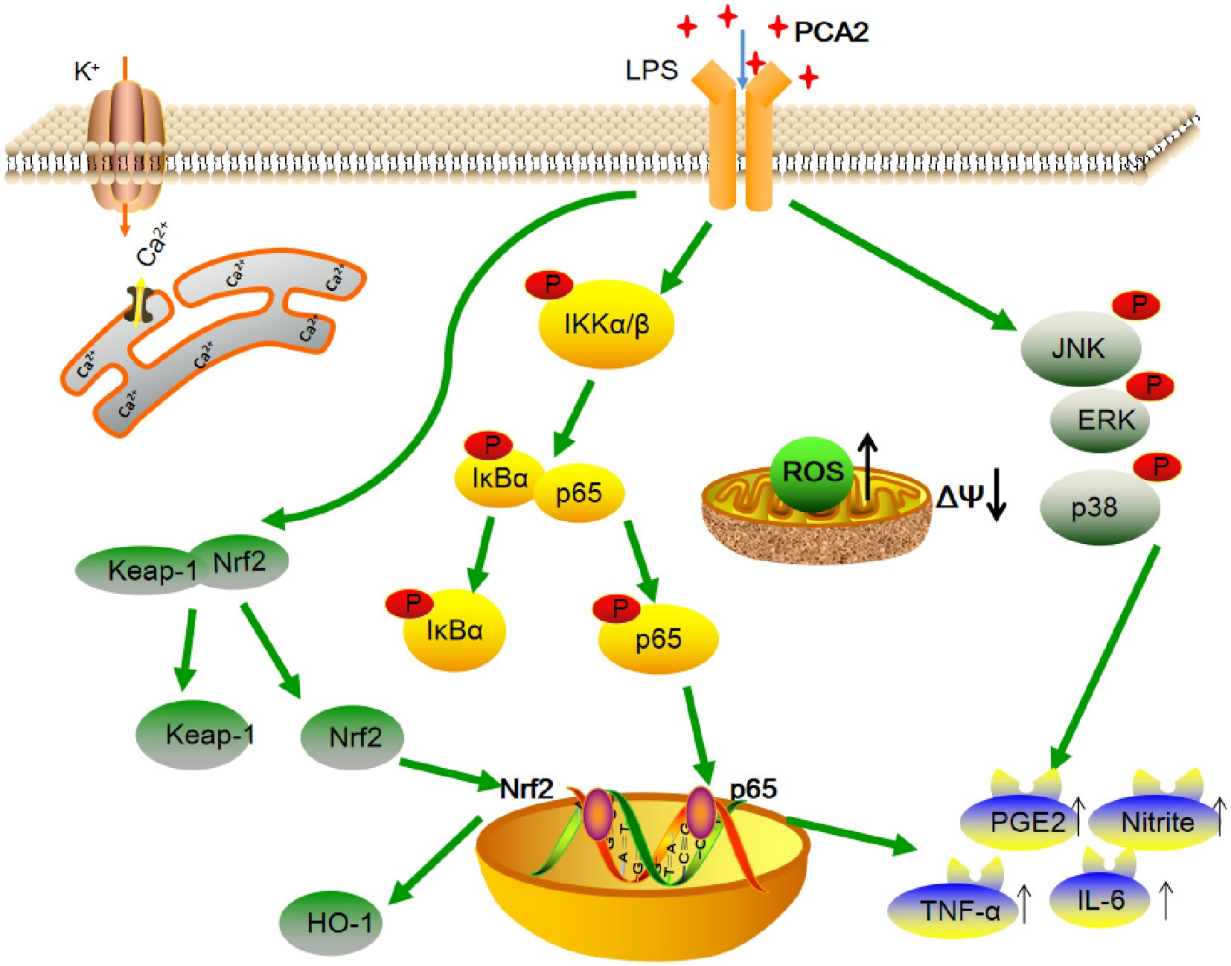
The schematic diagram of the mechanism of PCA2’s anti-inflammatory and anti-oxidative effects. PCA2 has an anti-inflammatory and anti-oxidant effect on LPS-induced RAW264.7 cells. The potential mechanisms involved in suppressing the NF-κB, MAPK, and Nrf2 pathway though decreases ROS generation, NO and Ca^2+^ exclusion, and the release of pro-infammatory cytokines such as IL-6, TNF-α and PGE2.

From the above, our study showed that PCA2 displays anti-inflammatory anti-oxidative effects through NF-κB, MAPK, Nrf2 pathways (Fig 8). Thus, PCA2 is expected to become a new drug for the clinical treatment of inflammation.

## Supporting information

S1 Fig PCA2 shows no detriment on RAW264.7 cells. Cells were treated with PCA2 (20, 40, 80 μM) for 24 h, except for the control group. Determination of nitrite levels by the Griess assay (n = 5).
(TIF)

S1 Raw images (PDF)
