# Supplementary figures and images for: Procyanidin A2, a polyphenolic compound, exerts anti-inflammatory and anti-oxidative activity in lipopolysaccharide-stimulated RAW264.7 cells
Source: PLoS One. 2020 Aug 5;15(8):e0237017. doi: 10.1371/journal.pone.0237017 (PMC7406031; doi:10.1371/journal.pone.0237017)

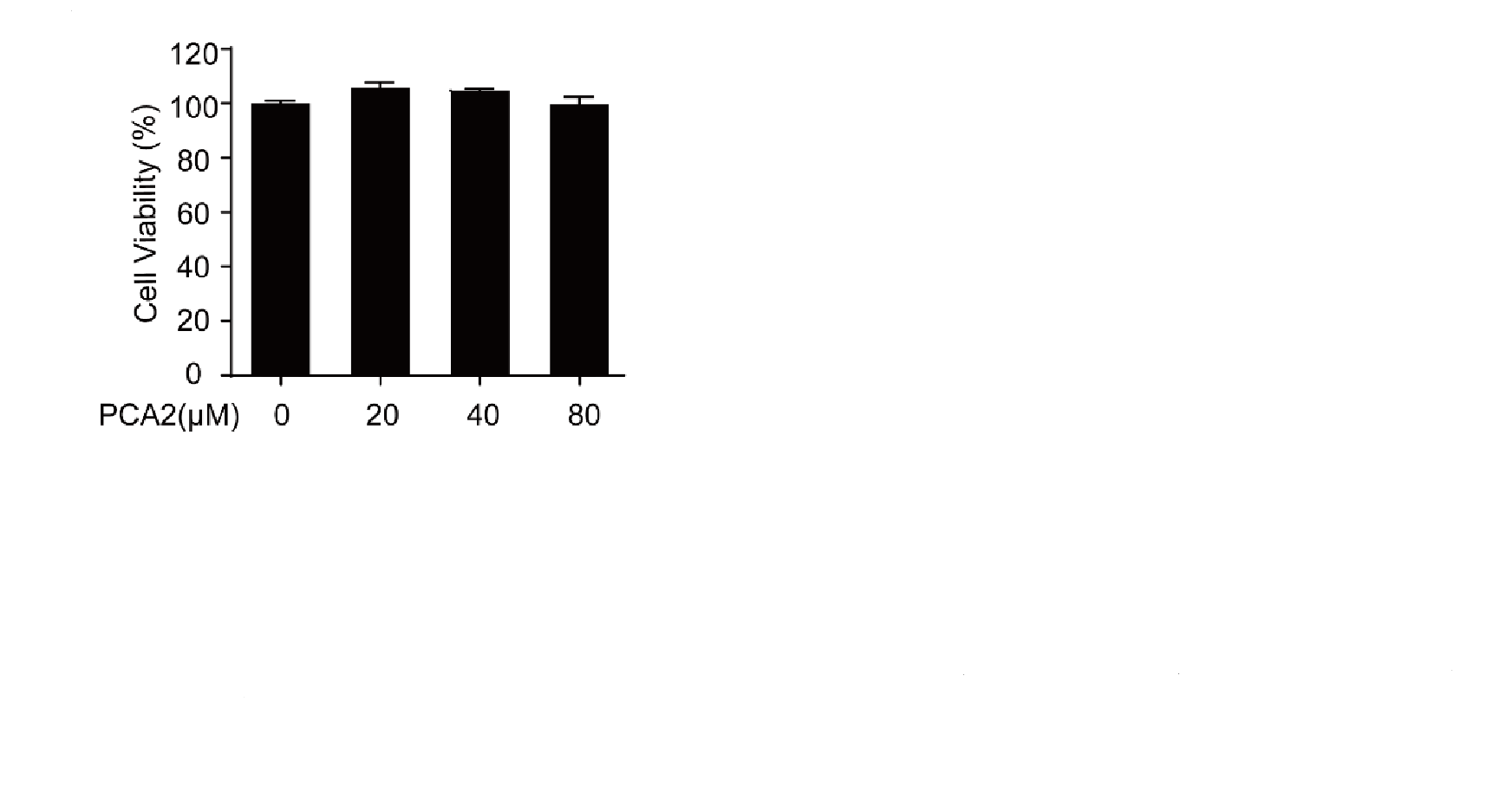

Supplement: S1 Fig — Cells were treated with PCA2 (20, 40, 80 μM) for 24 h, except for the control group. Determination of nitrite levels by the Griess assay (n = 5). (TIF) [file pone.0237017.s001.tif]

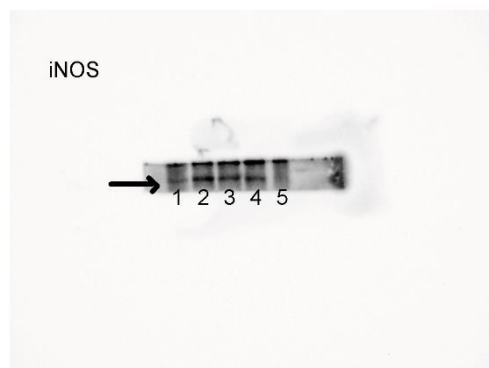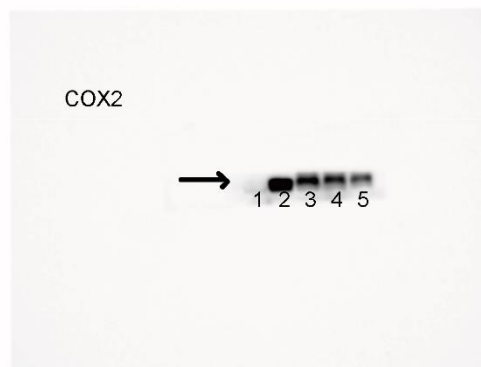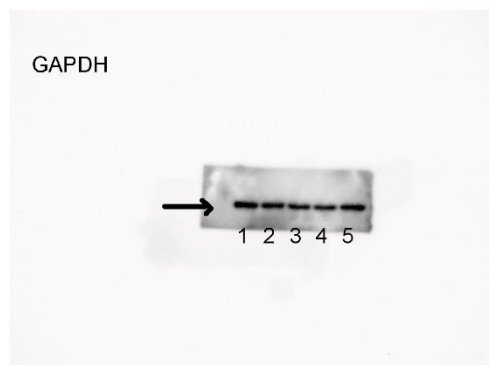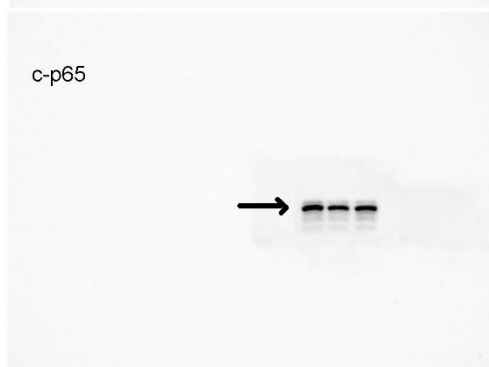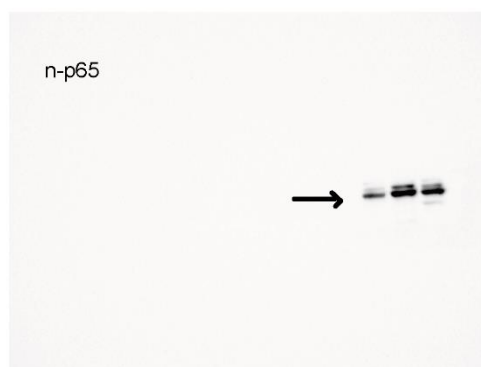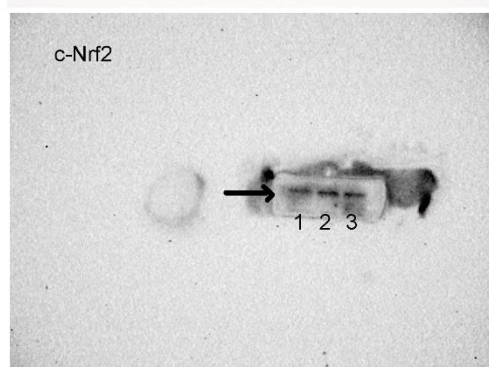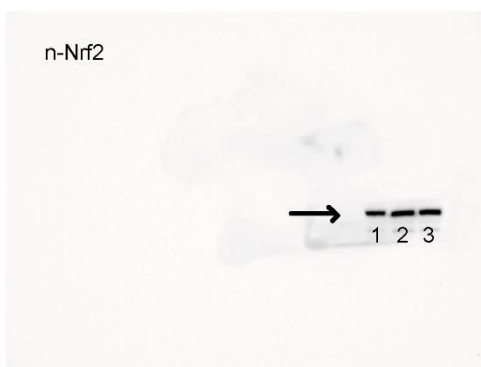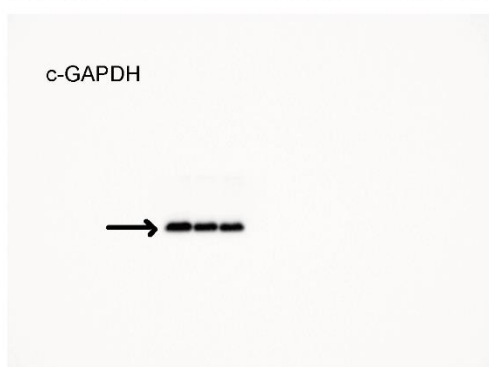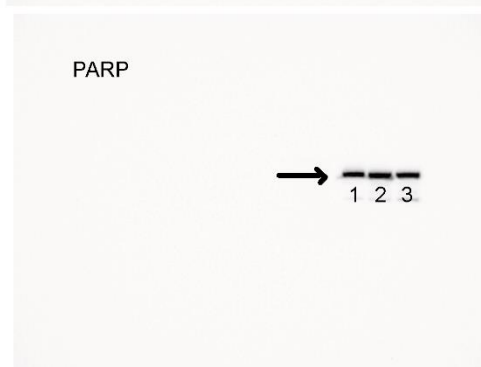

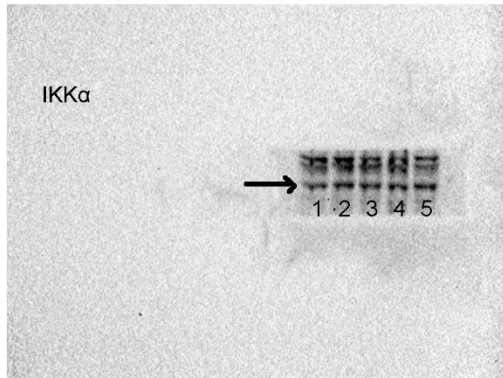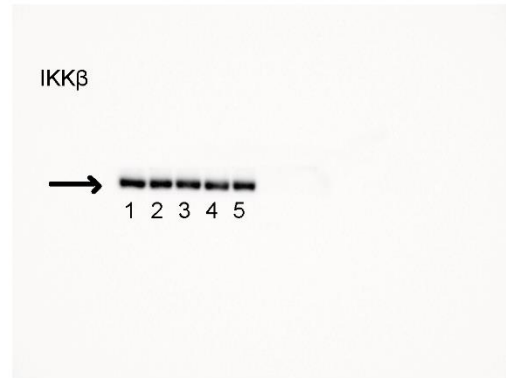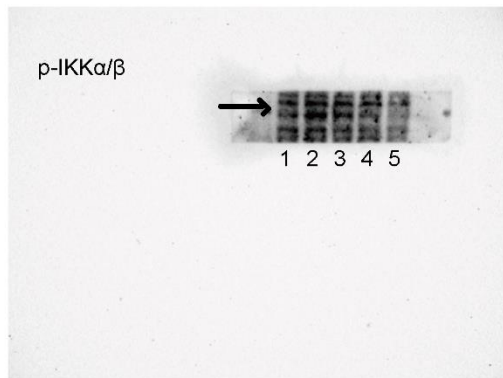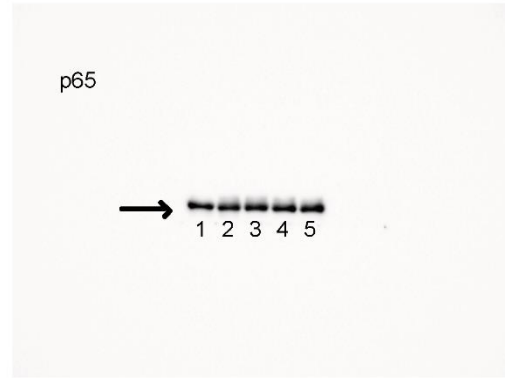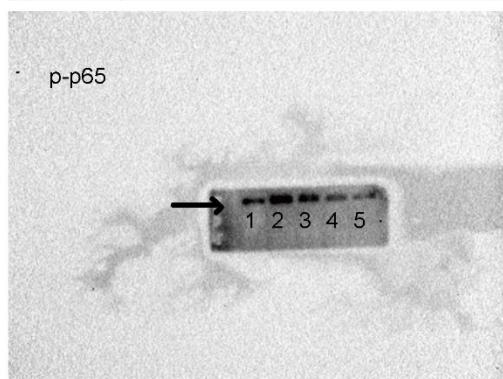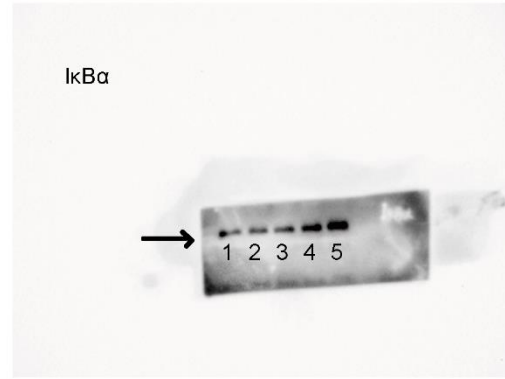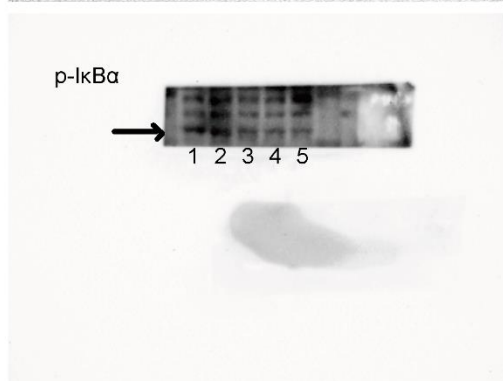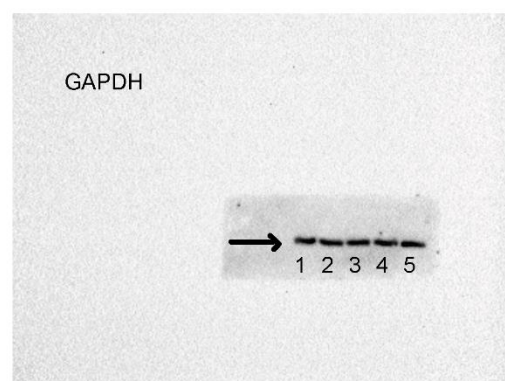

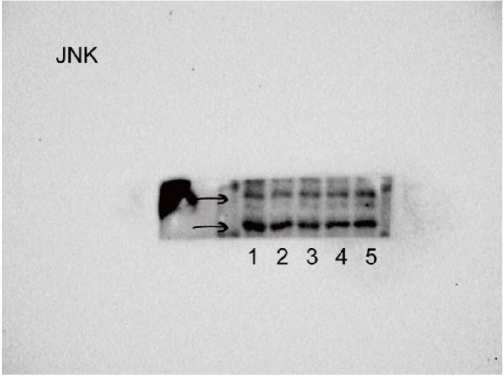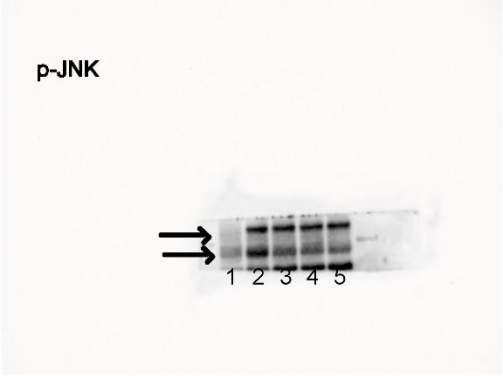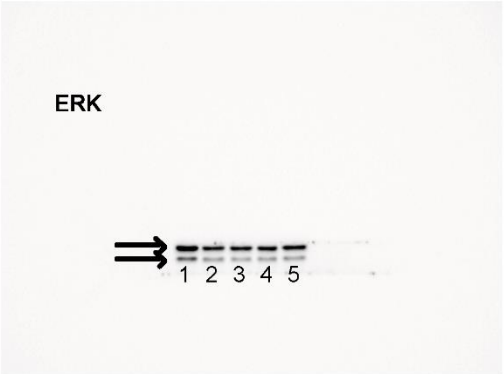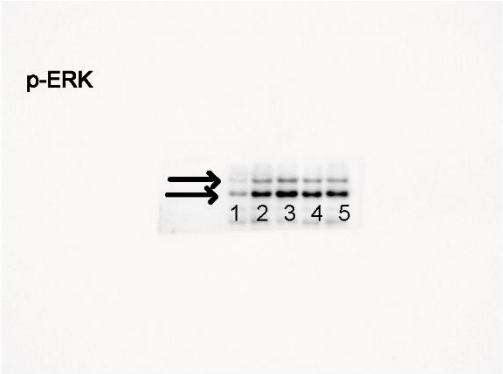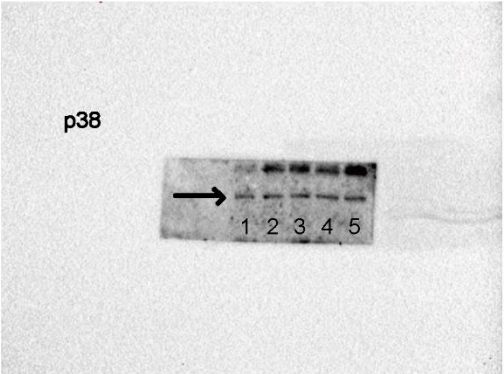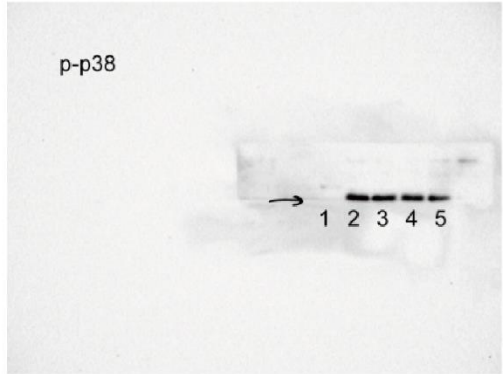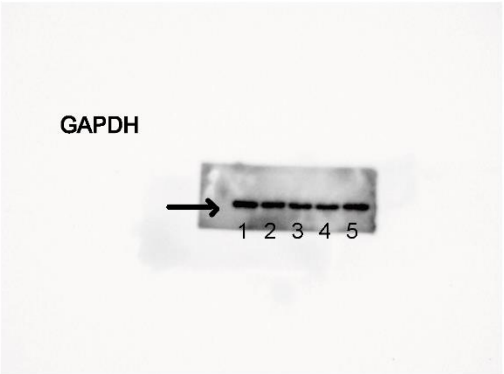

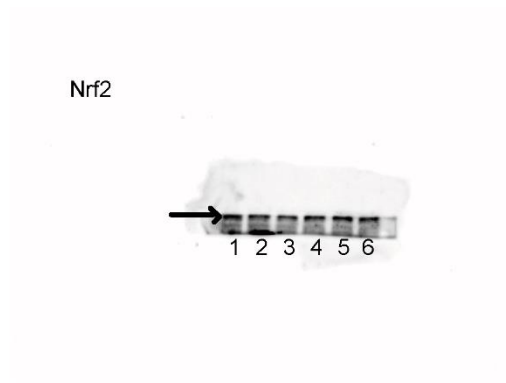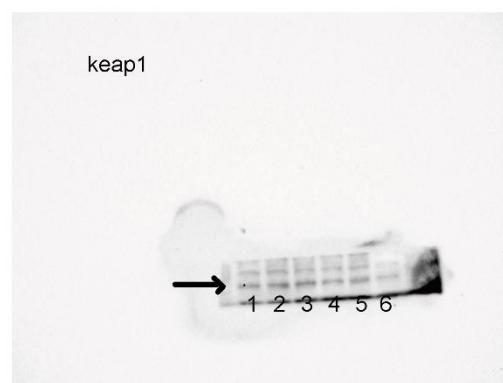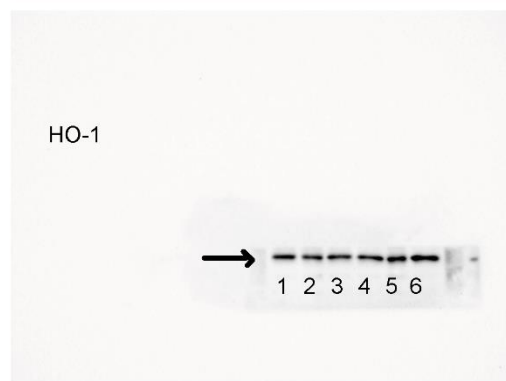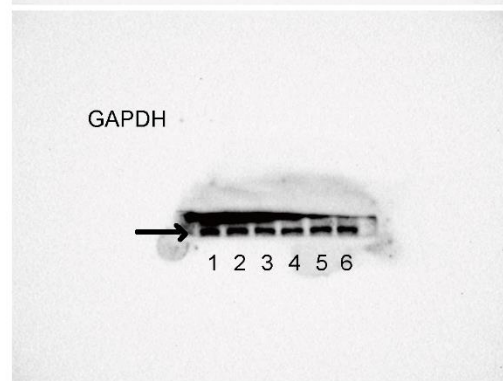

Supplement: S1 Raw images — (PDF) [file pone.0237017.s002.pdf]
